# Geography, Host Genetics, and Cross‐Domain Microbial Networks Structure the Skin Microbiota of Fragmented Brazilian Atlantic Forest Frog Populations

**DOI:** 10.1002/ece3.7594

**Published:** 2021-06-18

**Authors:** Anat M. Belasen, Maria A. Riolo, Molly C. Bletz, Mariana L. Lyra, L. Felipe Toledo, Timothy Y. James

**Affiliations:** ^1^ Department of Ecology and Evolutionary Biology University of Michigan Ann Arbor MI USA; ^2^ Center for Complex Systems University of Michigan Ann Arbor MI USA; ^3^ Department of Biology University of Massachusetts Boston Boston MA USA; ^4^ Instituto de Biociências Universidade Estadual Paulista Rio Claro Brazil; ^5^ Laboratório de História Natural de Anfíbios Brasileiros Departamento de Biologia Animal Instituto de Biologia Universidade Estadual de Campinas Campinas Brazil

**Keywords:** amphibian, Brazil's Atlantic Forest, microbial networks, skin microbiome

## Abstract

The host‐associated microbiome plays a significant role in health. However, the roles of factors such as host genetics and microbial interactions in determining microbiome diversity remain unclear. We examined these factors using amplicon‐based sequencing of 175 *Thoropa taophora* frog skin swabs collected from a naturally fragmented landscape in southeastern Brazil. Specifically, we examined (1) the effects of geography and host genetics on microbiome diversity and structure; (2) the structure of microbial eukaryotic and bacterial co‐occurrence networks; and (3) co‐occurrence between microeukaryotes with bacterial OTUs known to affect growth of the fungal pathogen *Batrachochytrium dendrobatidis* (*Bd*). While bacterial alpha diversity varied by both site type and host MHC IIB genotype, microeukaryotic alpha diversity varied only by site type. However, bacteria and microeukaryote composition showed variation according to both site type and host MHC IIB genotype. Our network analysis showed the highest connectivity when both eukaryotes and bacteria were included, implying that ecological interactions may occur among domains. Lastly, anti‐*Bd* bacteria were not broadly negatively co‐associated with the fungal microbiome and were positively associated with potential amphibian parasites. Our findings emphasize the importance of considering both domains in microbiome research and suggest that for effective probiotic strategies for amphibian disease management, considering potential interactions among all members of the microbiome is crucial.

## Graphical abstract

We use a natural laboratory to examine the relationship between host genetic diversity, bacterial diversity, and microeukaryote diversity in the skin microbiome of a Brazilian frog. We find that host immunogenetic diversity is associated with variation in microbiome diversity and structure, and present results that support novel associations between microeukaryotes and bacteria on frog skin. Our results have implications for the relationships between host genetics and health mediated through microbiome diversity and structure.

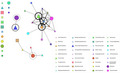

## 1 BACKGROUND

The host‐associated microbiome has recently captured the attention of wildlife disease researchers seeking to understand and predict disease‐associated wildlife declines. In particular, skin microbiome research is burgeoning in the field of amphibian disease. A majority of amphibian disease studies focus on chytridiomycosis caused by the pathogenic fungus *Batrachochytrium dendrobatidis* (*Bd*). *Bd* has been linked to severe amphibian declines around the world since at least the 1970s (Olson et al., 2013; Scheele et al., 2019; Lips et al., 2006; Carvalho et al., 2017). In some regions, however, no declines have been observed despite the presence of *Bd*. For example, plethodontid salamanders in the Eastern United States showed no evidence of disease‐associated declines despite the presence of *Bd* in the environment (Muletz et al., 2014). In a series of foundational studies, many of which were performed in vitro, the presence of certain bacteria cultured from salamander skin was correlated with reduced disease risk (Harris et al., 2006, 2009; Muletz et al., 2012). Further studies showed that this was also the case in some anurans (Lam et al., 2010; Harris et al., 2009; Vredenburg et al., 2011) and pointed to antifungal bacterial metabolite production as the main mechanism behind this correlation (Myers et al., 2012; Woodhams et al., 2014; Harris et al., 2009). These findings among others gave rise to interest in characterizing amphibian skin microbiome bacteria as a correlate of *Bd* susceptibility, and in using “probiotic strategies” (manipulating amphibian skin bacteria) to mitigate disease‐associated amphibian declines in the wild (Woodhams et al., 2014; Bletz et al., 2013; Voyles et al., 2018; Piovia‐Scott et al., 2017).

However, the potential nontarget impacts of manipulating bacteria are difficult to predict, as much remains to be understood about the diversity and assembly of the overall amphibian skin microbiome aside from the intensively studied *Bd* inhibitory bacteria. In particular, little is known about the ecological roles of nonbacterial taxa (but see, Kueneman et al., 2016; Kearns et al., 2017), or interactions between bacteria and skin microbial eukaryotes other than *Bd*. A diversity of microeukaryotes including fungi, microscopic metazoans, and protists have been identified on amphibian skin using high‐throughput sequencing (Belasen et al., 2019; Kueneman et al., 2017). In previous studies, fungi comprised the dominant eukaryotic taxon on adult amphibians (Kueneman et al., 2016) and exhibited greater efficacy in Bd inhibition compared with bacteria (Kearns et al., 2017). Although little is known about the ecological roles of these fungi in the amphibian skin microbiome, symbiotic fungi are known to aid in protection against fungal pathogens in other host‐microbial systems (Gao et al., 2010; Newsham et al., 1995). Fungi in the amphibian skin microbiome may also serve as hyperparasites, that is, parasites of pathogens/parasites. For example, the cryptomycete fungus *Rozella* parasitizes chytrid fungi (Gleason et al., 2012). Less is known about the symbiotic roles of host‐associated protists, although microbiome eukaryotes on the whole have been shown to impact health (Hoffmann et al., 2014; Holler et al., 2014) and immune function (Graham, 2008) in mammals. Thus, microbiome eukaryotes may significantly impact disease susceptibility in vertebrates, including amphibians. Without an understanding of the interactions between microbiome eukaryotes and bacteria, it is impossible to predict the potential microbiome‐wide effects of proposed *Bd* control measures that involve manipulating microbiome bacteria.

In addition, few studies to date have examined the genetic mechanisms that determine host‐associated microbiome assembly and diversity. From research on mammals, it is known that microbiome assembly and diversity can covary with overall host genetic diversity (Benson et al., 2010) as well as host immunogenetics (Marietta et al., 2015; Blekhman et al., 2015), with the latter relationship hypothetically resulting from interactions between immune cells and microbes including commensals and pathogens. In amphibians, previous studies have demonstrated that geography, host identity, and developmental stage can influence skin microbiome diversity (Walke et al., 2014; Griffiths et al., 2018; Kueneman et al., 2014). Yet only a single study to date has linked amphibian skin microbiome diversity with overall host genetic variability (Griffiths et al., 2018). Although no studies have directly examined the relationship between immunogenetics and microeukaryote diversity or structure in amphibians, an experimental study on the laboratory model frog *Xenopus laevis* suggested that MHC (major histocompatibility complex) immunogenes may determine the ability of hosts to tolerate different microbes (Barribeau et al., 2012). The relationship between immunogenes and the amphibian host‐associated microbiome remains to be explored and is increasingly relevant for wild amphibian populations threatened by emerging disease.

In a number of amphibian species, genetic diversity has been compromised due to anthropogenic habitat fragmentation (Allentoft & O’Brien, 2010). Although it is unknown to what extent habitat fragmentation impacts the amphibian skin microbiome, genetic erosion in fragmented amphibian populations has been observed at neutral loci as well as immunogenetic regions (Belasen et al., 2019) which may have implications for microbiome structure (Blekhman et al., 2015). In addition, fragmentation may cause a decline in microbial transmission, which in turn may alter microbial interactions and networks in host‐associated microbiomes (Rebollar et al., 2016; Adair & Douglas, 2017). However, the effects of habitat fragmentation on wildlife are subject to time lags (Tilman et al., 1994); genetic erosion resulting from inbreeding may not be detectable for several generations following habitat fragmentation, making the impacts on genetics and related factors difficult to detect in recently fragmented populations. Historically fragmented populations offer an opportunity to examine the effects of genetic erosion on the microbiome and broader animal health.

We evaluated the effects of long‐term habitat fragmentation on the amphibian skin microbiome using a historically fragmented model system in the Brazilian Atlantic Forest. This system consists of dozens of land‐bridge islands, which were naturally separated from the mainland 12,000–20,000 years ago via sea‐level rise (Suguio et al., 2005) and thus represent ancient forest fragments. Contemporary insular frog populations were once part of contiguous coastal populations and are now functionally isolated to the islands (Duryea et al., 2015; Bell et al., 2012). Using this geographic setting, we examined the impacts of geography and host genetics on skin microbiome diversity and community structure in a single frog species, *Thoropa taophora* (Cycloramphidae), found across coastal mainland and island sites. The island populations of *T. taophora* have experienced fragmentation‐induced genetic erosion at both neutral and immunogenetic loci (Duryea et al., 2015; Belasen et al., 2019). Previous work also showed that island and coastal mainland populations exhibited low *Bd* prevalence and very low zoospore loads, suggesting low *Bd* susceptibility in this species (Belasen et al., 2019). Commonly, it is hypothesized that low *Bd* susceptibility is associated with the presence of anti‐*Bd* microbes. Therefore, this system offered the opportunity to ask a number of important questions about the relationships between geography, host genetics, bacteria, and eukaryotes in the skin microbiome, within the context of potential protection from *Bd*.

We used amplicon‐based high‐throughput DNA sequencing to analyze bacterial and eukaryotic microbes found in skin swab samples collected from *T. taophora* frogs across coastal mainland and island sites. We examined the relationships between microbes, geography, and genetics, as well as the connections of microbes across domains (bacteria versus eukaryotes). We compared bacteria we recovered from *T. taophora* skin swabs to a database of amphibian microbiome bacterial isolates that have been previously tested for anti‐*Bd* activity in challenge assays (Woodhams et al., 2015). Because the mechanism by which these bacteria inhibit *Bd* is not specific to the interaction, but works through metabolites produced by bacteria that have broad antifungal activity (Harris et al., 2009), this database may be used as a proxy for antifungal inhibitory compound production. We used this database to identify which of the bacteria on *T. taophora* skin matched bacterial OTUs that were previously identified as *Bd* inhibitory, *Bd* enhancing, and having no effect on *Bd*, and evaluated whether these categories explained co‐occurrence patterns between these bacteria and (non‐*Bd*) microeukaryotes found in the *T. taophora* microbiome. Our study was designed to address the following research questions: (a) Does geography and/or host genetic diversity structure the microbiome community? (b) How is bacterial diversity and community assembly related to microeukaryotic diversity and community assembly in the skin microbiome? (c) Do bacteria that affect *Bd* growth have predictable associations with other skin microeukaryotes that could result in unintended consequences if probiotic anti‐*Bd* bacteria are applied to frog skin?

## 2 METHODS

### 2.1 Study system and field sampling

**FIGURE 1:**
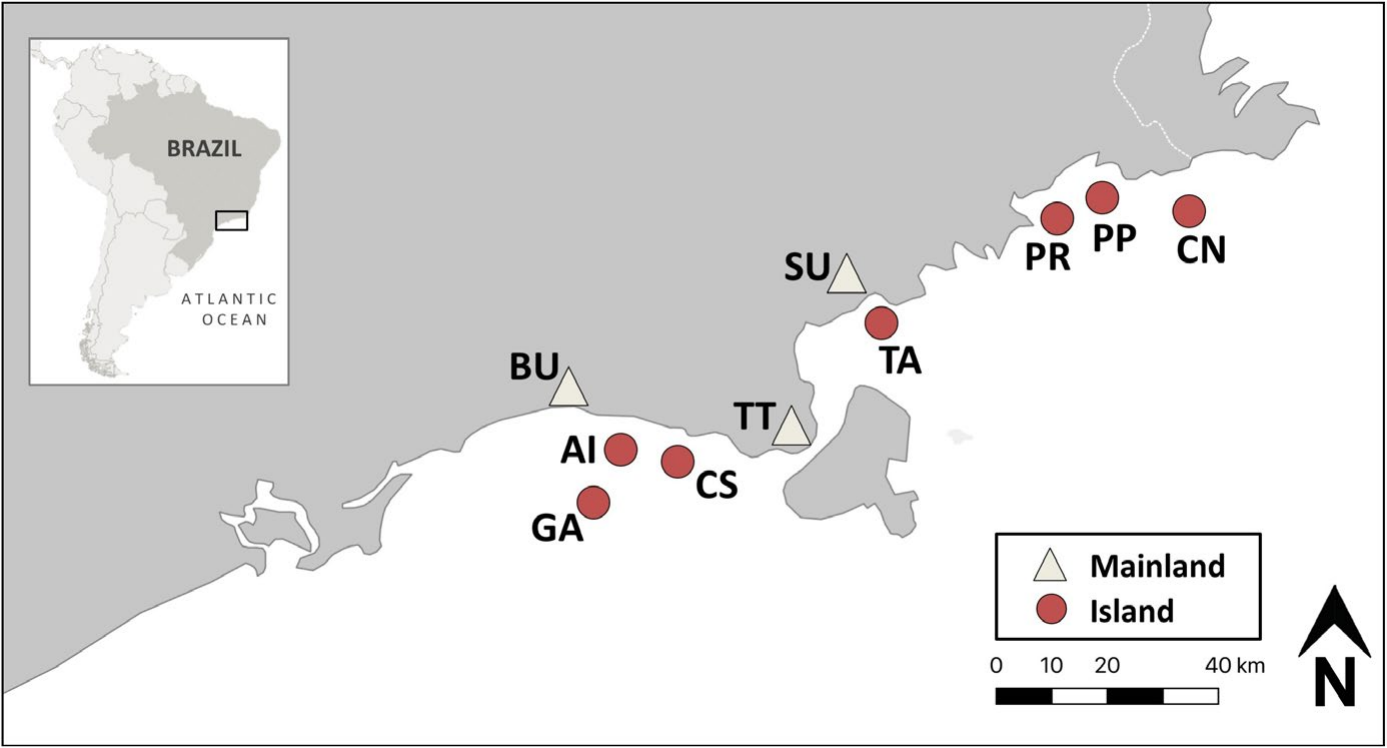
Locations of *Thoropa taophora* sampling sites on the coast and islands of São Paulo, Brazil. Red circles indicate islands, and white triangles indicate coastal (mainland) sites. Site information including full site names, two‐letter codes, latitude/longitude, and frog sample size can be found in Table 1

The focal species for this study is *Thoropa taophora*, a cycloramphid frog with a unique tolerance for coastal habitat that allows a wide distribution across the coastal Atlantic Forest of São Paulo State (Duryea et al., 2008). Adult *T. taophora* frogs (*n* = 175 total) were sampled from each of ten study populations: seven island populations and three coastal mainland populations (Figure 1, Table 1; SISBio collection permit 27745‐13). Genetic diversity is lower in island *T. taophora* populations relative to coastal mainland populations, both at neutral (microsatellite) loci (Duryea et al., 2015) as well as at the MHC IIB immunogenetic locus (Belasen et al., 2019). To examine how host genetics impact skin microbiome diversity, skin swab samples were analyzed from the same individuals that were previously genotyped at MHC IIB (see Belasen et al., 2019). Prior to tissue collection, frogs were thoroughly washed with sterile (autoclaved) distilled water and then swabbed on the ventral surface using standard protocols that minimize cross‐contamination (Bletz, Vences, et al., 2017). Swabs and tissue samples were stored in 70% ethanol in sterile microcentrifuge tubes before laboratory processing. DNA was extracted from swabs using a Qiagen DNeasy Blood and Tissue kit, and DNA extracts were stored at −20°C prior to further molecular work.

**TABLE 1:** Sampling site data

Site name	Site code	Site type	Latitude	Longitude	Sample size	MHC IIB heterozygosity, Ho	MHC IIB Allelic Richness, N_A_
As Ilhas	AI	Island	−23.789276	−45.711507	4	0	1
Couve Sul	CS	Island	−23.805592	−45.670011	7	0.43	2
Couves Norte	CN	Island	−23.422075	−44.854066	30	0.43	2
Gatos	GA	Island	−23.805592	−45.670011	3	0	1
Porcos Pequena	PP	Island	−23.377864	−44.904266	20	0	1
Prumirim	PR	Island	−23.384791	−44.945678	22	0.09	4
Tamanduá	TA	Island	−23.597168	−45.288857	25	0	1
Barra do Una	BU	Coastal	−23.761536	−45.770697	20	0	2
Sununga	SU	Coastal	−23.508867	−45.133827	20	0.7	11
Toque Toque	TT	Coastal	−23.835912	−45.509922	24	0.51	10

Sample size is the number of frogs collected at each site. MHC IIB heterozygosity is the observed heterozygosity, or number of heterozygotes over the total individuals genotyped from each population. Site locations are shown in Figure 1

### 2.2 Microbiome sequencing and bioinformatic processing

Individual swab DNA extracts were PCR‐amplified, pooled, and sequenced on the Illumina MiSeq platform (250 bp paired‐end reads) in two assays: (a) barcoded 16S primers 515F and 806R (Vences et al., 2016) were used to examine bacterial diversity; and (b) barcoded 18S v4 primers TAReuk454FWD1 and TAReukREV3 (Stoeck et al., 2010) were used to examine microeukaryote diversity. 16S libraries were constructed at the Universidade Estadual Paulista (BR) and sequenced at the Tufts University Core facility (USA) while 18S library preparation and sequencing were performed at the University of Michigan (USA). Negative (template‐free) controls were run simultaneously with each sequencing library to ensure there was no contamination from PCR or sequencing reagents.

Sequences were quality‐filtered and processed using the Quantitative Insights into Microbial Ecology (QIIME) MiSeq pipeline using default settings (Caporaso et al., 2010). As no mock community was included as a positive sequencing control, low abundance OTUs were filtered from the dataset using a conservative abundance threshold (<0.005% of all reads) (Bokulich et al., 2013). Sequences were clustered into operational taxonomic units (OTUs) using a 97% similarity threshold and compared against reference databases to assign taxonomy (GreenGenes 13.8 and RDP search for 16S, Silva 119 and BLAST search for 18S). Chimeras were identified and filtered using UCHIME2 (Edgar, 2016). 16S sequences from chloroplasts and mitochondria and 18S sequences assigned to frog or other nontarget nonmicrobial species (*e.g*., Streptophyta) were filtered from the dataset. 16S sequences were rarefied to 2000 per sample and 18S sequences were rarefied to 1000 per sample based on visual examination of read accumulation curves and plots of rarefaction values versus number of samples retained across sites (Figure S1). These sequence threshold values for rarefaction were selected to balance achieving an adequate representation of microbial communities with retaining sufficient site sample sizes, and are within the range of similar previous studies that have used 1000–2000 as threshold values for 16S v4 datasets (Bletz et al., 2016, 2017).

To determine whether potential ecological relationships between bacteria and microeukaryotes reflect potential ecological relationships between bacteria and *Bd*, bacterial OTU representative sequences from the *T. taophora* samples were compared against a reference sequence database of bacteria previously isolated from amphibian skin and categorized according to effects on *Bd* growth in co‐culture experiments (Woodhams et al., 2015). The BLAST algorithm was implemented, and an E‐value threshold of E < 1e‐20 was used to identify OTU matches with the reference database. Matching *T. taophora* skin bacteria were binned into categories as *Bd* enhancing, *Bd* inhibiting, or having no effect on *Bd* growth.

### 2.3 Data analysis

To evaluate overall patterns of microbiome alpha diversity in the rarefied 16S and 18S datasets, Spearman's correlation tests were implemented in R (vrs. 1.7–11; (R Core Team, 2018)) and performed between 16S and 18S alpha diversity according to (a) OTU richness and (b) phylogenetic diversity calculated in QIIME. To evaluate the relationships between alpha diversity and geography and host genetics, two‐way ANOVA tests were performed in R (vrs. 1.7–11; R Core Team, 2018). Separate two‐way ANOVA tests were run for each response variable with four ANOVAs run in total. The four response variables were calculated in QIIME and consisted of (a) OTU richness for bacteria (16S), (b) phylogenetic diversity for bacteria, (c) OTU richness for microeukaryotes (18S), and (d) phylogenetic diversity for microeukaryotes. Each response variable was tested against the factors of site type (island versus coastal mainland) and MHC IIB genotype (homozygote versus heterozygote). Two‐way ANOVA models were initially run for each response variable with an interaction between site type and MHC IIB genotype, and if the interaction term was non‐significant, the model was re‐run with an additive effect between factors instead. Assumptions of linear models were confirmed with visual examination of residuals versus fitted values plots and normal Q‐Q plots. Response variables were natural log‐transformed to meet assumptions of equal variance and normality. Abundance‐based diversity indices (*e.g*., Shannon's Indices) were not analyzed because using sequence reads as a proxy for abundance can be problematic due to primer amplification bias and variable copy number of 16S and 18S across microbial taxa (Bletz, Archer, et al., 2017; Song et al., 2018; Kembel et al., 2012; Větrovský & Baldrian, 2013).

To evaluate microbial community structure across geography and host population genetics, beta diversity was calculated using the unweighted (*i.e*., does not account for reads/sequence abundance) UniFrac (phylogeny‐based) method in QIIME. Bacterial and microeukaryotic beta diversity were analyzed in separate Mantel tests against geographic distance, neutral genetic distance (*F*_ST_ calculated from microsatellites), and immunogenetic distance (*F*_ST_ calculated from MHC IIB sequences) among populations. Mantel tests were implemented in the ade4 package of R (Dray & Dufour, 2007; Dray et al., 2007; Chessel et al., 2004; R Core Team, 2018).

To examine associations between microbial taxa and geography or host frog MHC IIB genotype, data were statistically analyzed and visualized using packages implemented in Python (vrs. 2.7.13) and using Matplotlib (Hunter, 2007; Rossum, 1995). Associations between microbial communities and geography or frog MHC IIB genotype were determined by simulating an expected null (randomized) distribution of host frog microbiomes. To create the null distribution, a two‐column data table was first created with column 1 being the site type (island or coastal) or MHC IIB genotype (heterozygous or homozygous) of a host frog and column 2 being a single microbial OTU found on that frog. After the data table was populated for all frogs and microbial OTUs in the dataset, column 2 (microbial OTU) was held constant while column 1 (site type or frog genotype) was shuffled randomly. This was repeated 1,000 times to create two sets of random microbial occurrence distributions, one for analysis of microbial associations with site type and a second for analysis of microbial associations with host frog genotype.

Co‐occurrence between microbial OTUs within and among domains (Bacteria versus Eukaryotes) was analyzed with a third null distribution of microbial communities. Because of potential effects of site on microbial presence and community structure (*e.g*., some microbes only co‐occur on frogs because the microbes themselves solely occur at the same subset of sampling sites) and site‐MHC IIB genotype interactions (as homozygotes and heterozygotes are not evenly distributed across sites or site types; Table 1), an expected null distribution of microbes accounting for site‐specific presence/absence of each microbe was created. This null distribution of microbes was achieved through within‐site randomization using MCMC edge swapping, a standard method for network datasets (Petersen, 1891; Besag & Clifford, 1989; Fosdick et al., 2018). This method allows any configuration to be reached from any starting point and allows for even sampling along all allowed states as forward and backward swaps are equally likely. To achieve this, first, two microbe–frog pairs were randomly selected, with each pair consisting of a single randomly selected microbial OTU found on a single randomly selected frog. Microbial OTUs were then swapped between the selected frogs when three criteria were met: (a) the frogs were different individuals with the same MHC IIB genotype (either both homozygous or both heterozygous); (b) the OTUs were different from one another; and (3) neither frog already hosted the microbe it would receive via the swap. Microbe swapping was performed with 1,000 repetitions for each frog–microbe pair to construct a single set of randomized frog–microbe pairs. The distribution of co‐occurrences under this null model was estimated using 320,000 such randomized sets.

To test whether hypothesized bacterial effects on *Bd* extend to diverse microeukaryotic members of the microbiome, bacterial OTUs that matched the Woodhams et al. (2015) database were binned according to their hypothesized ecological significance with regard to *Bd* (*Bd* inhibitory, *Bd* enhancing, or no effect on *Bd*). The co‐occurrences of bacteria within each category with microbiome eukaryotes were then compared with the third null distribution of microbial OTUs.

For all microbial association/co‐occurrence analyses, the probability of non‐random microbial association/co‐occurrence (*p*) was calculated by comparing observed versus expected (null/randomized) counts of microbial association/co‐occurrence. *P*‐values were evaluated at significance levels of α = 0.05 and 0.01with correction applied to account for multiple comparisons (Benjamini et al., 1995). Using the results of the tests of co‐occurrences within and among all microbial taxa, microbial networks for bacteria only, microeukaryotes only, and for all bacteria and microeukaryotes were visualized using Matplotlib (Hunter, 2007; Rossum, 1995).

## 3 RESULTS

### 3.1 Overall patterns of microbiome diversity

**FIGURE 2:**
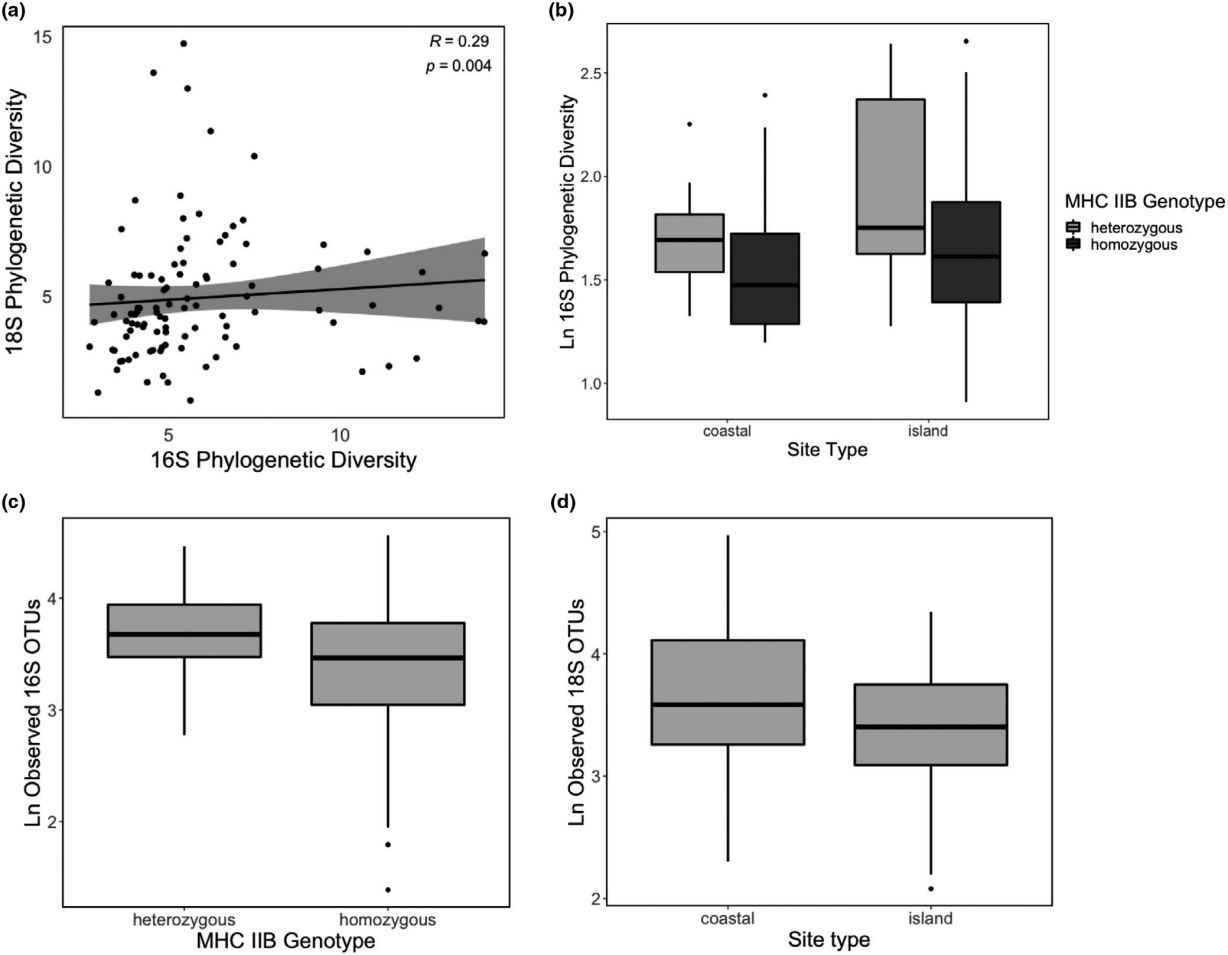
Microbiome alpha diversity. (a) Phylogenetic diversity of bacteria and eukaryotes on Thoropa taophora skin were significantly positively correlated. (b) Bacterial phylogenetic diversity varied significantly across both site type and host frog MHC IIB genotypes. (c) Bacterial OTU richness varied significantly across host frog MHC IIB genotypes. (d) Microeukaryotic OTU richness varied significantly across site types

There were 303 bacterial OTUs and 845 microeukaryotic OTUs recovered across all samples after filtering and rarefaction. Bacterial phylogenetic diversity was positively correlated with microeukaryotic phylogenetic diversity across all samples (Spearman's rank correlation, ρ = 0.28, *p* <.01; Figure 2a). There was no significant correlation between bacterial and microeukaryotic OTU richness (Spearman's rank correlation, *p* >.05).

**FIGURE 3:**
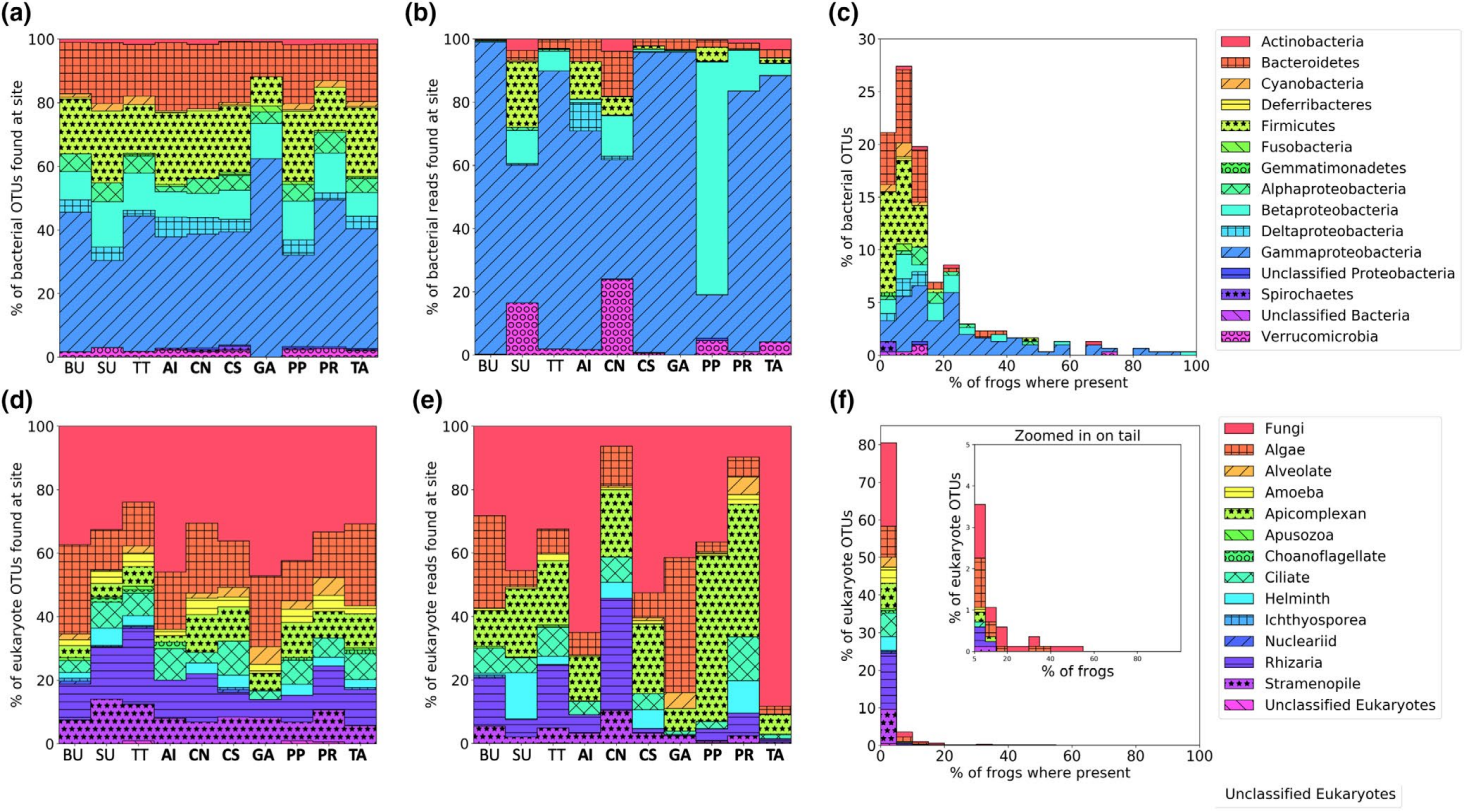
Distribution of microbiome taxonomic diversity across study sites and individuals. Bacteria are shown in a‐c and eukaryotes in D‐F. Stacked barplots show the relative abundance of microbial taxa across sites by number of OTUs (a and d) and reads (b and e). Bolded site codes indicate island sites. Frequency histograms (c and f) show the percent of OTUs across individual host frogs. In C, solid indigo bars represent Proteobacteria, which show a signature consistent with a core bacterial microbiome as these are present across the majority of frogs. In f, there is not a clear signature of a core eukaryotic microbiome, as no taxa extend across the majority of individuals. Fungi come closest as a set of fungal OTUs are found on ~50% of individual frogs (f inset)

Proteobacteria, particularly Gammaproteobacteria, were the most dominant bacterial taxon across all samples, both by number of OTUs and sequence reads (Figure 3a,b). Gammaproteobacteria also formed a core bacterial microbiome (*i.e*., this taxon was abundant across samples; Figure 3c). Among the eukaryotic microbiota, fungi were dominant by both number of OTUs and sequence reads (Figure 3d,e). No core group of eukaryotic taxa was recovered, though some fungal OTUs were common and found in approximately 50% of samples (Figure 3f). These common fungal OTUs were members of the Ascomycota, Basidiomycota, and unclassified fungi.

### 3.2 Associations between geography, host genetics, and the skin microbiome

Patterns of alpha diversity across site types and MHC IIB genotypes varied among bacteria and microeukaryotes. In all analyses, there were no statistically significant interactions between site type and MHC IIB genotype, and all response variables (16S OTU richness, 16S PD, 18S OTU richness, 18S PD) were natural log‐transformed to meet the assumptions of ANOVA tests. Bacterial phylogenetic diversity (PD) varied by both MHC IIB genotype and by site type (two‐way ANOVA, *F*(2, 117) = 4.536, *p* =.01, site type *p* <.05, MHC IIB genotype *p* =.01; Figure 2b). Average bacterial PD among island frogs was 6.11 compared with average PD of 5.25 for coastal mainland frogs, while average bacterial PD among MHC IIB heterozygotes was 6.64 compared with an average of 5.58 in homozygotes. Microeukaryotic PD did not vary by either site type or MHC IIB genotype (two‐way ANOVA, *F*(2, 139) = 2.587, *p* >.05). Bacterial OTU richness varied only by MHC IIB genotype (two‐way ANOVA, *F*(2, 117) = 3.289, *p* <.05, site type *p* >.05, MHC IIB genotype *p* =.01; Figure 2c) with MHC IIB heterozygotes hosting 43.0 bacterial OTUs on average compared with 34.3 average bacterial OTUs on MHC IIB homozygotes. In contrast, microeukaryotic alpha diversity varied by only site type for OTU richness (two‐way ANOVA, *F*(2, 139) = 5.062, *p* <.01, site type *p* <.01, MHC IIB genotype *p* >.05). On average, mainland coastal frogs hosted 48.1 microeukaryotic OTUs compared with 32.6 average microeukaryotic OTUs on island frogs.

**FIGURE 4:**
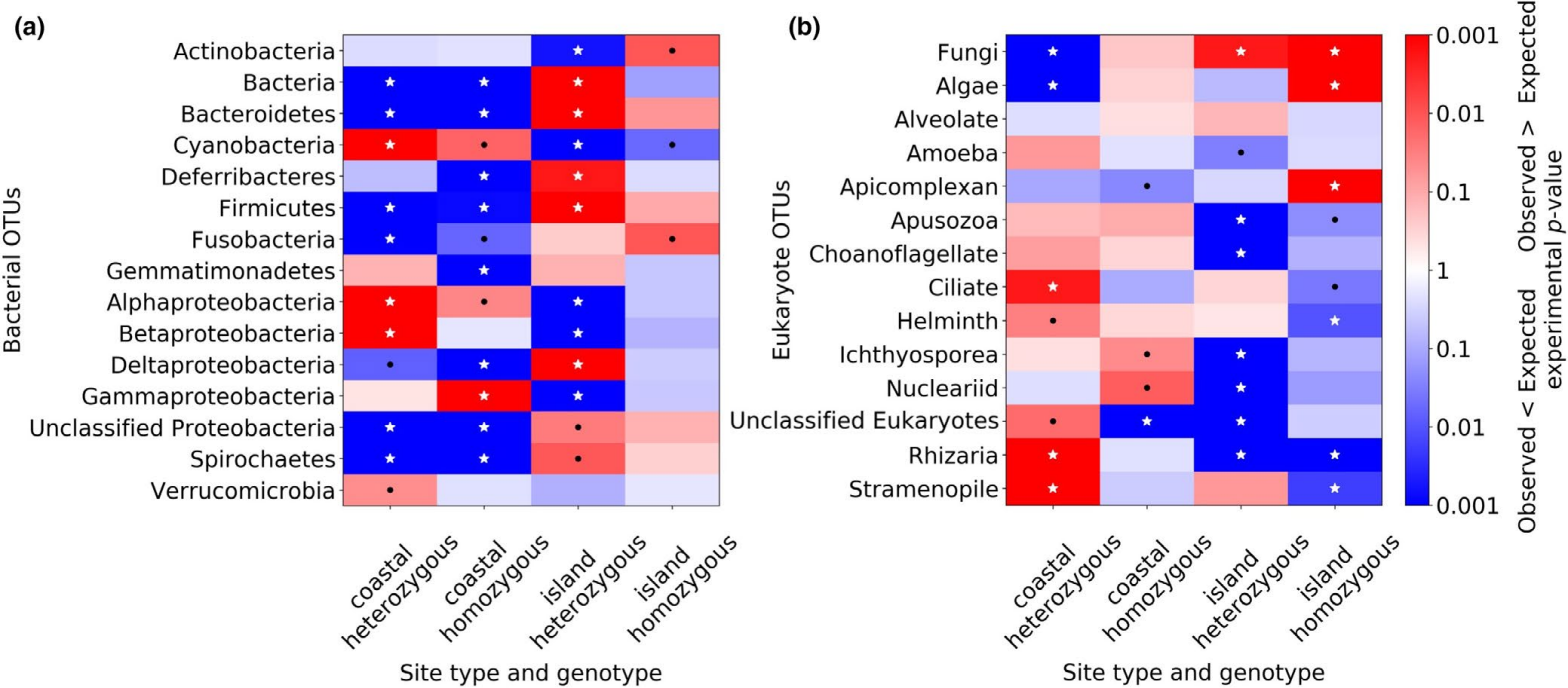
Heat maps of skin microbes across site type and host MHC IIB genotype for bacteria (a) and eukartyoes (b). Associations between intersecting factors of microbial OTUs and site type (coastal/mainland versus. island) and MHC IIB genotype (heterozygous versus. homozygous) are shown in each column. The more saturated the red, the stronger the positive association between taxa and site type or genotype, and the more saturated the blue, the stronger the negative association. To determine associations, actual distribution of microbes was compared with 1,000 randomly generated microbiomes within each site. Black dots represent significant deviation from random expectations with *p* <.05, and white stars represent *p* <.01

Community composition showed variable patterns. Beta diversity did not vary by geographic distance or either measure of genetic distance (microsatellite F_ST_ and MHC IIB F_ST_) according to Mantel tests (all *p* >.05). However, when OTUs were compared with random expectations, significant associations with site type and MHC IIB genotype were observed for both bacteria and microeukaryotes (Figure 4). Among the bacteria, Cyanobacteria and Alphaproteobacteria showed significant positive associations with coastal mainland sites regardless of host MHC IIB genotype, while four bacterial groups (Bacteroidetes, Firmicutes, Fusobacteria, unclassified Proteobacteria, and Spirochaetes) showed positive associations with island sites regardless of genotype (Figure 4a). Among the microeukaryotes, fungi were significantly positively associated with island sites regardless of host genotype (Figure 4b). Coastal mainland MHC IIB heterozygotes showed significant positive associations with Ciliates, Helminths, unclassified microeukaryotes, Rhizaria, and Stramenopiles, while mainland homozygotes were significantly positively associated with Ichthyosporea and Nucleariids. Island MHC IIB homozygotes showed significant positive associations with Algae and Apicomplexans.

### 3.3 Microbial networks within and among domains

Separate networks were constructed for bacteria and microeukaryotes based on tests of co‐occurrence between OTUs within and among taxonomic groups across domains (Figure S2). A dominant bacterial network assembled that consisted of 9/16 bacterial taxa: Bacteroidetes, Firmicutes, and Deltaproteobacteria were at the center of the formed network, with connections to Deferribacteres, Fusobacteria, Spirochaetes, Verrucomicrobia, unclassified Proteobacteria, and unclassified bacteria (Figure S3). The remaining groups did not form any connections, although there were strong connections formed among OTUs within the Gammaproteobacteria. Within the microeukaryotes, no network connections formed among the 21 taxonomic groups, but there were significant connections between OTUs within the Algae and Rhizaria (Figure S4).

**FIGURE 5:**
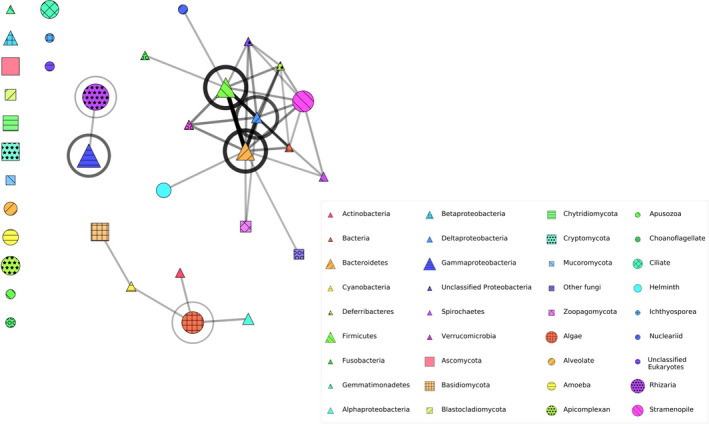
Microbiome network showing associations between bacterial and microeukaryotic OTUs. Bacteria are represented by triangles, fungi are represented by squares, and protists (non‐fungal microeukaryotes) are represented by circles. The size of the symbol indicates the relative abundance of each taxon by number of OTUs. Stronger associations are indicated by thicker/darker network branches between symbols (associations of OTUs among taxa) or circles around symbols (associations of OTUs within a taxon). Links were included between taxa which co‐occurred significantly (a = 0.05) more often than in the null model. A circle around a taxon represents a self‐edge. The edge weight was scaled according to the Z‐score of the co‐occurrence (observed ‐ expected) / standard deviation, to the power of 0.5 to make the variation in score more visually clear

The cross‐domain network analysis resulted in a number of previously unconnected taxa becoming connected with others, with 4/6 previously unconnected bacterial groups and 8/21 previously unconnected eukaryotic groups becoming connected to taxa across domains (Figure 5). Three networks formed, the largest of which consisted of the previously constructed bacteria‐only network with additional connections between bacterial taxa and six eukaryotic taxa: two fungal groups (Zoopagomycota and unclassified fungi) and three protist groups (Helminths, Nucleariids, and Stramenopiles). The second largest network formed between three bacterial groups and two eukaryotic groups, all five of which had previously been unconnected in the bacteria‐only and eukaryote‐only networks: Actinobacteria, Cyanobacteria, Alphaproteobacteria, Basidiomycota, and Algae. The third and smallest network consisted of Gammaproteobacteria and Rhizaria.

### 3.4 Associations between microbiome eukaryotes and bacteria reported to inhibit, enhance, or have no effect on *Bd* growth

**FIGURE 6:**
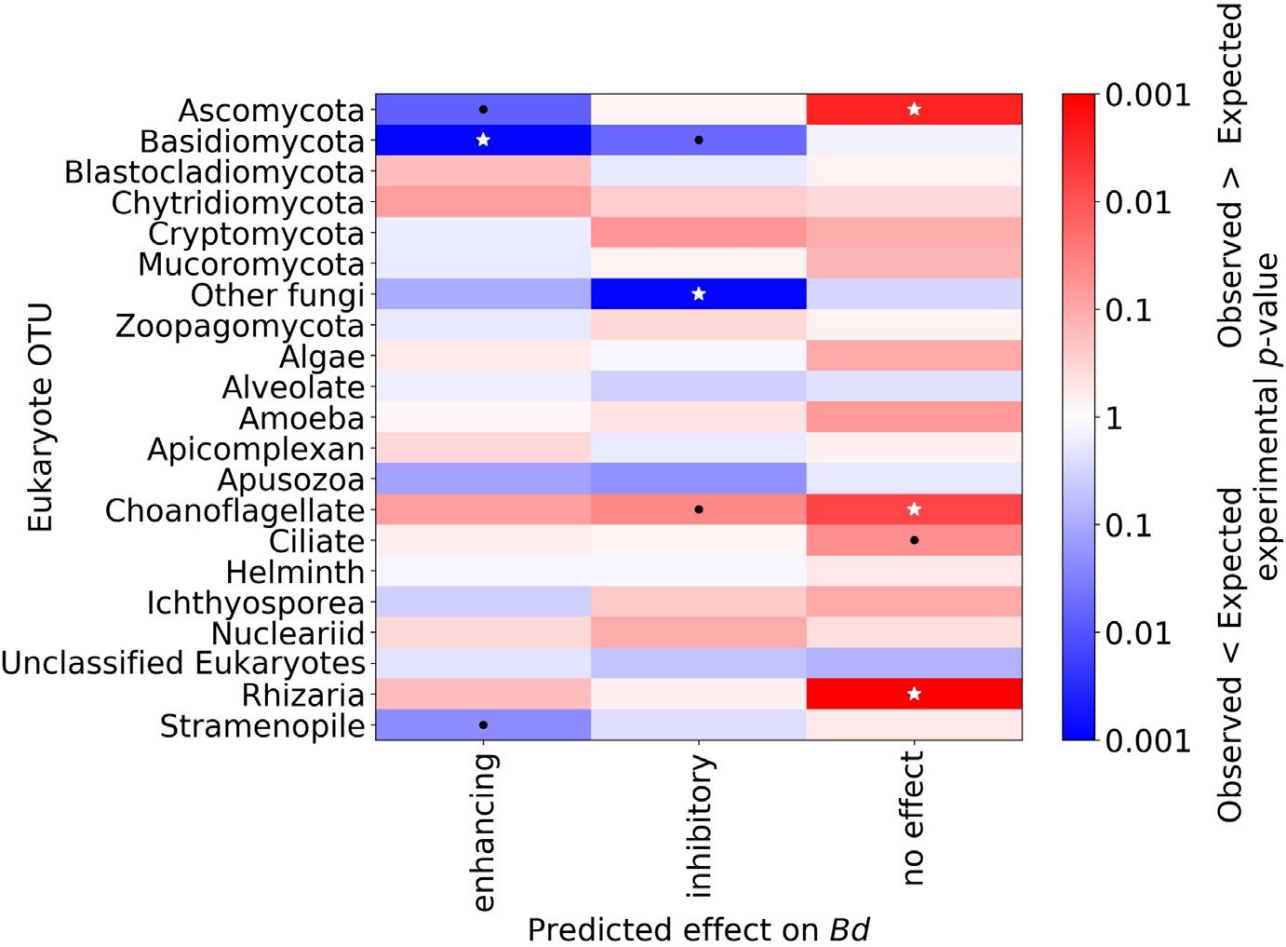
Heat map of microeukaryote co‐occurrence with bacterial OTUs found in *T. taophora* skin swabs. Bacteria are binned into groups corresponding to “Predict effect on *Bd*” based on matches to bacterial OTUs categorized by Woodhams et al. (2015). The more saturated the red, the stronger the positive association between two taxa, and the more saturated the blue, the stronger the negative association. Black dots represent a significant deviation from random co‐occurrence with *p* <.05, and white stars represent *p* <.01

When compared to bacterial OTUs that had been previously tested against Bd in co‐culture inhibition experiments (Woodhams et al., 2015), nearly half (45%) of *T. taophora* skin bacterial OTUs showed a match at the BLAST E‐value threshold of E < 1e‐20 (Figure S5). Tests of co‐occurrence between eukaryote groups and these matched bacterial OTUs revealed that enhancing, inhibitory, and no effect do not generally reflect the associations of these bacteria with fungi specifically or microeukaryotes generally (Figure 6). *Bd*‐enhancing bacteria were significantly negatively associated with the Ascomycota and Basidiomycota fungi as well as Stramenopiles. *Bd* inhibitory bacteria showed significant positive associations with the Choanoflagellates and significant negative associations with the Basidiomycota fungi and other unclassified fungi. These also showed nonsignificant positive associations with Cryptomycota fungi, Ichthyosporeans, and Nucleariids, and negative associations with Apusozoa. Finally, bacteria that were previously found to have no effect on *Bd* were significantly positively associated with Ascomycota fungi, Choanoflagellates, Ciliates, and Rhizaria.

## 4 DISCUSSION

### 4.1 Amphibian skin microbiomes exhibited high microeukaryote diversity and were dominated by Proteobacteria

In this study, we examined amphibian skin microbiome structure and diversity with respect to geography and host genetics. In analyzing both bacterial and microeukaryote OTUs, we recovered microbial associations with geographic and host genetic factors, as well as unexpected patterns of microbial co‐occurrence across domains. The diversity of microeukaryotes we recovered is higher than previous reports from wild frogs: We recovered 845 OTUs in our study compared with, for example, 255 OTUs on *Rana cascadae* (Kueneman et al., 2017) and 500 OTUs on *Anaxyrus boreus* (Bletz et al., 2013). In contrast, the level of bacterial diversity we recovered is lower than previous reports: We recovered 303 bacterial OTUs compared with ~600 OTUs on *Rana italica* (Větrovský & Baldrian, 2013), although we note that this could be due to different filtering thresholds. Our recovery of bacteria from 11 phyla is within the range of taxonomic diversity previously recovered from amphibian skin, with for example 10–18 bacterial phyla reported from three species (McKenzie et al., 2012). Our analysis showed that total microeukaryotic and bacterial phylogenetic diversity were positively correlated across all samples, which is a novel finding to our knowledge.

Proteobacteria, and in particular Gammaproteobacteria, was the most dominant bacterial phylum on *T. taophora* skin across all study populations, in terms of both OTUs and relative abundance (Figure 3). This is similar to findings from bacterial microbiome studies of other tropical post‐metamorphic anurans (Harris, Brucker, et al., 2009; Kueneman et al., 2019; Abarca et al., 2018; Varela et al., 2018; Bletz et al., 2017). Proteobacteria are known to be common in a variety of environments and contain bacteria that can be pathogenic in amphibians (Hill et al., 2010). The dominance of Proteobacteria on amphibian skin has been hypothesized to result from a protective symbiosis between bacteria and amphibians, as many members of the Proteobacteria produce anti‐*Bd* metabolites (Brucker et al., 2008; Becker, 2015). The presence of a high number of Proteobacteria on *T. taophora* skin could potentially contribute to its low apparent susceptibility to *Bd* (Belasen et al., 2019). It is important to note however that the present study is correlative; without experimental manipulations, it is difficult to pinpoint which factors (*e.g*., the physiology of the skin, mucosal biochemistry, host‐microbial evolutionary processes, or interactions with the saline coastal environment) are responsible for the overwhelming dominance of Proteobacteria on *T. taophora* skin.

Although bacteria were less diverse than microeukaryotes in our samples, bacteria could nevertheless dominate the skin microbiome according to microbial biomass, which we did not quantify in our study. Sequence reads are sometimes used to estimate relative abundance, but this has been shown to be an unreliable measure due to known sequencing biases among microbial taxa (Amend et al., 2010). It is possible that taxa representing fewer OTUs (*i.e*., bacterial species/strains) represent a higher proportion of microbial biomass, and this should be considered in interpretations of our results. We recommend that future research to address the relationship between microbial diversity and abundance employ high‐throughput sequencing alongside quantitative analyses, for example, quantitative PCR.

### 4.2 Microbiome diversity and structure varied with site type and host immunogenetics

Immunogenotype at the MHC IIB locus was associated with alpha diversity of bacteria on *T. taophora* skin such that MHC IIB heterozygotes hosted a greater number of bacterial OTUs and higher bacterial phylogenetic diversity. Site type (*i.e*., island versus coastal mainland) was also a significant factor in alpha diversity for both bacteria and microeukaryotes (although only for microeukaryote OTU richness, not for phylogenetic diversity). However, beta diversity was not associated with geographic distance or genetic structure of populations at either neutral genetic markers or the MHC IIB immunogenetic locus. These results differ from previous studies on amphibians, in which there were similarly no geographic effects on amphibian skin microbiome structure, but there was a significant association with metapopulation genetic structure (Griffiths et al., 2018; Hernández‐Gómez et al., 2017). One possible explanation for the discrepancy between our results and the results from previous studies (barring host identity factors) is that our study populations represent a set of connected mainland populations contrasted with a set of island populations that have been isolated for 12,000–20,000 years. The lack of association with genetic differentiation in our populations may be due to this relatively long period of divergence relative to other studies, isolation between island sites resulting in different environmental availability of microbes, or simply environmental differences between island and mainland sites.

As microbial diversity was lower in island frogs, this suggests that microbiome diversity may be influenced by genetic diversity: Island populations are genetically impoverished and possess lower microeukaryotic and bacterial diversity relative to coastal mainland populations. Unlike in bacteria, microeukaryotic alpha diversity was not statistically associated with MHC IIB genotype. However, several microeukaryote groups showed significant associations with MHC IIB genotype. Interestingly, MHC IIB homozygotes showed significant positive associations with potential amphibian parasites including Ichthyosporeans in coastal mainland populations, and fungi and Apicomplexans in island populations. These patterns may indicate associations between genetic factors and assembly of the microbiome such that MHC IIB molecules mediate the prevalence of beneficial microbes in addition to pathogenic ones.

Taken together, our results imply that host genetics, and specifically MHC IIB genotype, may play a significant role in determining overall microbiome diversity and structure. Although MHC genotype is thought to primarily associate with immune defense against pathogens, results from laboratory and field studies suggest that MHC genotype and allelic composition can impact amphibian host‐associated microbial assemblages more broadly (Harris, Lauer, et al., 2009; Hernández‐Gómez et al., 2018). The positive associations we found between MHC IIB heterozygosity and bacterial microbiome diversity, as well as with overall microbiome community composition, suggest there may be unknown relationships between MHC molecules and host‐associated microbes beyond antagonistic interactions between immune molecules and pathogens. However, further research is needed to confirm these associations in other species and determine the contributing mechanisms.

### 4.3 Cross‐domain co‐occurrence in the amphibian skin microbiome network

Our microbiome network analyses revealed a number of notable patterns. When analyzed separately, the bacterial network consisted of one major group, while no microeukaryote groups formed significant connections with one another. However, in the overall microbial network, a number of microbial groups exhibited cross‐domain co‐occurrence: a majority of previously unconnected bacterial groups (4/6) and a number of previously unconnected microeukaryote groups (8/21) became connected in the overall microbiome networks. To our knowledge, ours is the first study to demonstrate these positive cross‐domain network connections in the amphibian skin microbiome.

One important implication of this result is that previously undocumented ecological interactions may exist between microbiome bacteria and eukaryotes that in turn may significantly impact microbial assembly. It is currently unclear how widespread cross‐domain associations are, as previous studies that have examined both bacteria and microeukaryotes on amphibian skin have focused on taxon‐specific associations, namely between *Bd* inhibitory bacteria and fungi (Kueneman et al., 2016), or between *Bd* and either bacteria or microeukaryotes (Kueneman et al., 2017). While potential antagonistic interactions with *Bd* have been the focus in cross‐domain research on the amphibian skin microbiome, microbial interactions can occur across the spectrum of biological symbioses (reviewed in (Deveau et al. 2018)). Mutualistic interactions between bacteria and microeukaryotes have been documented in other systems; for example, mycorrhizae‐helper bacteria are known to indirectly facilitate plant–fungal interactions in the multitrophic mycorrhizal complex (Frey‐Klett & Garbaye, 2005). An alternative explanation for our network analysis results is that bacteria and eukaryotes positively co‐occur due to cofiltering via specific host, environmental, or other exogenous factors unrelated to microbial interactions. Further research is needed on cross‐domain microbial co‐occurrence patterns, microbial interactions, and implications for amphibian host health.

### 4.4 *Bd* inhibitory and enhancing bacteria have variable effects on microbiome fungi and protists

Our dataset included a number of bacteria previously shown to inhibit *Bd*, which have been generally termed “antifungal” (Vences et al., 2016). However, bacteria with previously demonstrated effects on *Bd* growth did not show general patterns with *T. taophora* skin microbiome fungi or other microeukaryotes. As might be expected, bacteria previously found to enhance *Bd* growth were positively associated with the Chytridiomycota, although *Bd* was not present in our 18S dataset. However, *Bd*‐enhancing bacteria were negatively associated with Ascomycota and Basidiomycota fungi as well as Stramenopiles. Perhaps more critical are the relationships with *Bd* inhibitory bacteria, as these have been proposed for use in probiotic treatments for *Bd* management (Walke & Belden, 2016; Bletz et al., 2013). *Bd* inhibitory bacteria showed significant negative associations with Basidiomycota fungi and other unclassified fungi in the *T. taophora* skin microbiome. *Bd* inhibitory bacteria were also positively associated with Choanoflagellates and showed positive although nonsignificant associations with the Zoopagomycota and Ichthyosporea.

These associations demonstrate the importance of understanding potential effects of probiotics on the amphibian skin microbiome and consequently on amphibian health. For example, specific attempts to increase *Bd* inhibiting bacteria and/or reduce *Bd‐*enhancing bacteria in wild frog populations could reduce fungi in the Dikarya (Ascomycota and Basidiomycota), some of which are known to benefit amphibian health (Kearns et al. 2017), and/or augment poorly studied parasites such as Ichthyosporea protists (Rowley et al., 2013) and Zoopagomycota fungi (Seyedmousavi et al., 2015; Badali et al., 2010) as well as Choanoflagellates that are known to be parasitic in other aquatic ectotherm hosts (Kerk et al., 1995). These hypothetical effects warrant further study, for example through culture‐based or in vivo challenges between proposed probiotic bacteria and these potentially impacted microeukaryotes. It bears noting that the apparently low susceptibility to *Bd* observed in *Thoropa taophora* (Belasen et al., 2019) may indicate that the results from this study may not apply to more *Bd*‐susceptible amphibian species. Nonetheless, our findings demonstrate the importance of understanding cross‐domain interactions and microbiome stability as it relates to amphibian health when considering probiotic treatments in wild populations. Evaluation of cross‐domain interactions and relationships should be part of the decision‐making process in determining whether to employ probiotic conservation strategies.

### 4.5 Limitations and future research priorities

Taken together with recent studies (Kueneman et al., 2017; Kearns et al., 2017), our results suggest that focusing only on bacteria provides an incomplete picture of the host‐associated microbiome. Granted, as in many other amphibian microbiome studies (McKenzie et al., 2012) our study presents microbes at a relatively coarse phylogenetic resolution. Very large differences in ecology and environmental requirements likely exist between OTUs within higher‐order classification levels, and the patterns we detected may change with higher‐resolution taxonomic data. With advancing technology allowing for increased sequence length (*e.g*., third‐generation sequencing), more efficient microbiome analysis pipelines (*e.g*., QIIME2), and higher quality reference sequence databases, future cross‐domain microbiome research at higher taxonomic resolution should be prioritized.

Our results imply that host genetic diversity and MHC IIB genotype play a role in structuring the amphibian skin microbiome. However, we acknowledge that differences in microbiome diversity and structure among site types and MHC IIB genotypes could be due to a number of factors other than or in addition to host genetics. Variation in the microbiome among site types could be explained by differences in environmental filtering in coastal versus island sites, island isolation favoring longer‐dispersing microbes, or alternatively by unexplored host factors (*e.g*., diet (Antwis et al., 2014)). Additional research is warranted to quantify the relative contributions of host factors, environmental factors, and other variables that contribute to microbiome diversity and structure.

Our network analyses suggest that there may be important interactions between bacteria and microeukaryotes that have been missed by previous microbiome studies focusing on only one microbial domain or specific microbial interactions. Given the widespread use of bacterial probiotic treatments in humans as well as in domesticated and wild animals (Cheng, 2017; Ghadban, 2002; Gram et al., 1999) and the interest in expanding these strategies to wild amphibians (Walke & Belden, 2016), future studies should prioritize advancing our understanding of interactions between microbiome bacteria and eukaryotes.

## CONFLICT OF INTEREST

The authors have no conflicts of interest to declare.

## AUTHOR CONTRIBUTION

**Anat M Belasen:** Conceptualization (lead); Formal analysis (equal); Funding acquisition (equal); Investigation (equal); Methodology (equal); Visualization (equal); Writing‐original draft (lead). **Maria A Riolo:** Formal analysis (equal); Visualization (equal); Writing‐review & editing (equal). **Molly Bletz:** Formal analysis (equal); Methodology (equal); Validation (equal); Visualization (equal); Writing‐review & editing (equal). **Mariana Lúcio Lyra:** Investigation (equal); Methodology (equal); Writing‐review & editing (equal). **Felipe Toledo:** Conceptualization (equal); Funding acquisition (equal); Investigation (equal); Project administration (equal); Writing‐review & editing (equal). **Tim James:** Conceptualization (equal); Investigation (equal); Project administration (equal); Supervision (lead); Writing‐review & editing (equal).

## Supporting information

Fig. S1

Fig. S2

Fig. S3

Fig. S4

Fig. S5

Supplementary materials

## Data Availability

Sequences were deposited in the NCBI Short Read Archive (18S sequences: Accession PRJNA720394; 16S sequences: Accession PRJNA720436). Samples were also registered in SISGEN (#A713DBD).
