# Supplementary figures and images for: Geography, Host Genetics, and Cross‐Domain Microbial Networks Structure the Skin Microbiota of Fragmented Brazilian Atlantic Forest Frog Populations
Source: Ecol Evol. 2021 Jun 18;11(14):9293–307. doi: 10.1002/ece3.7594 (PMC8293785; doi:10.1002/ece3.7594)

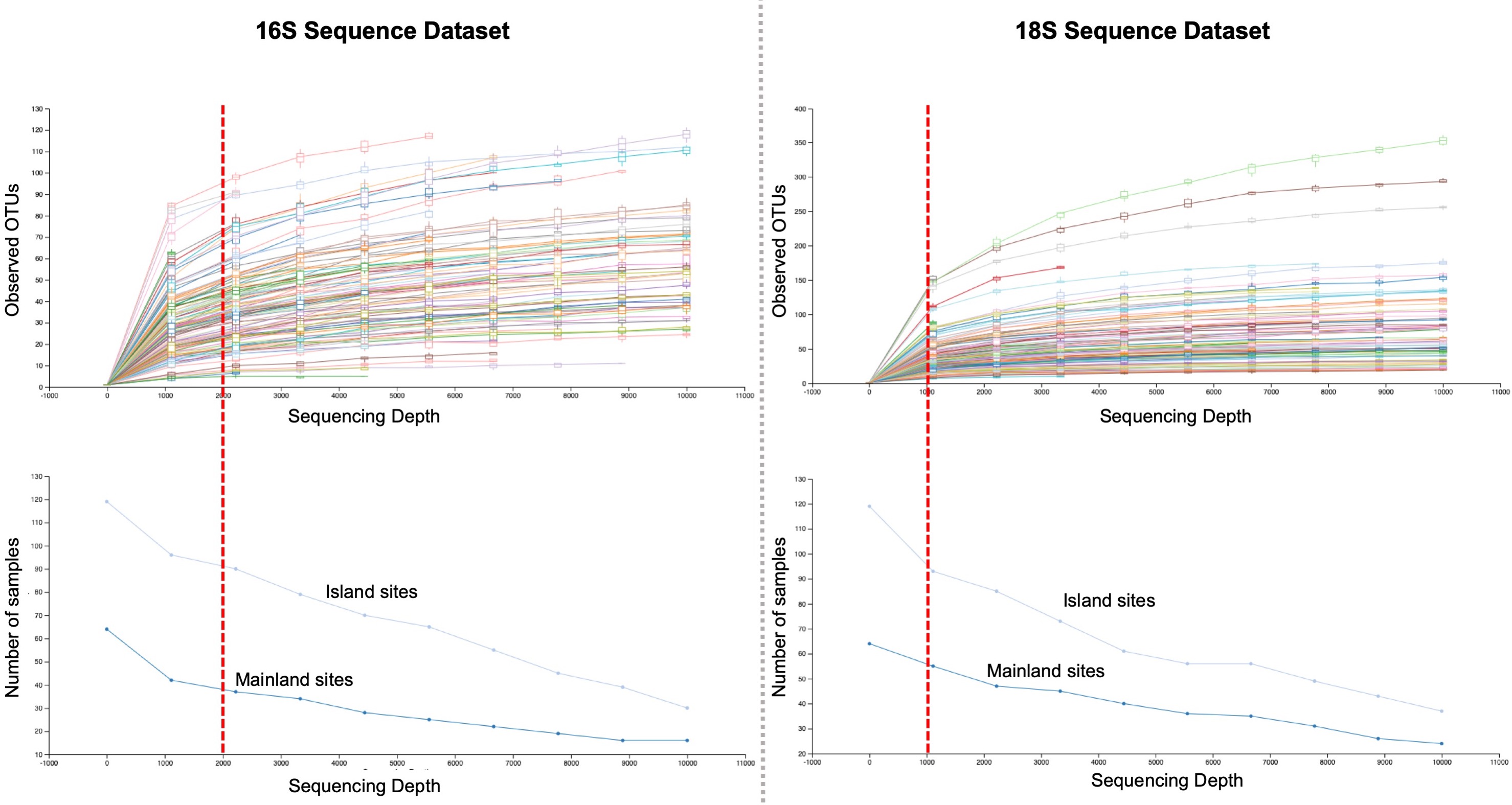

Supplement: Supplementary file 1 — Fig. S1 [file ECE3-11-9293-s006.jpg]

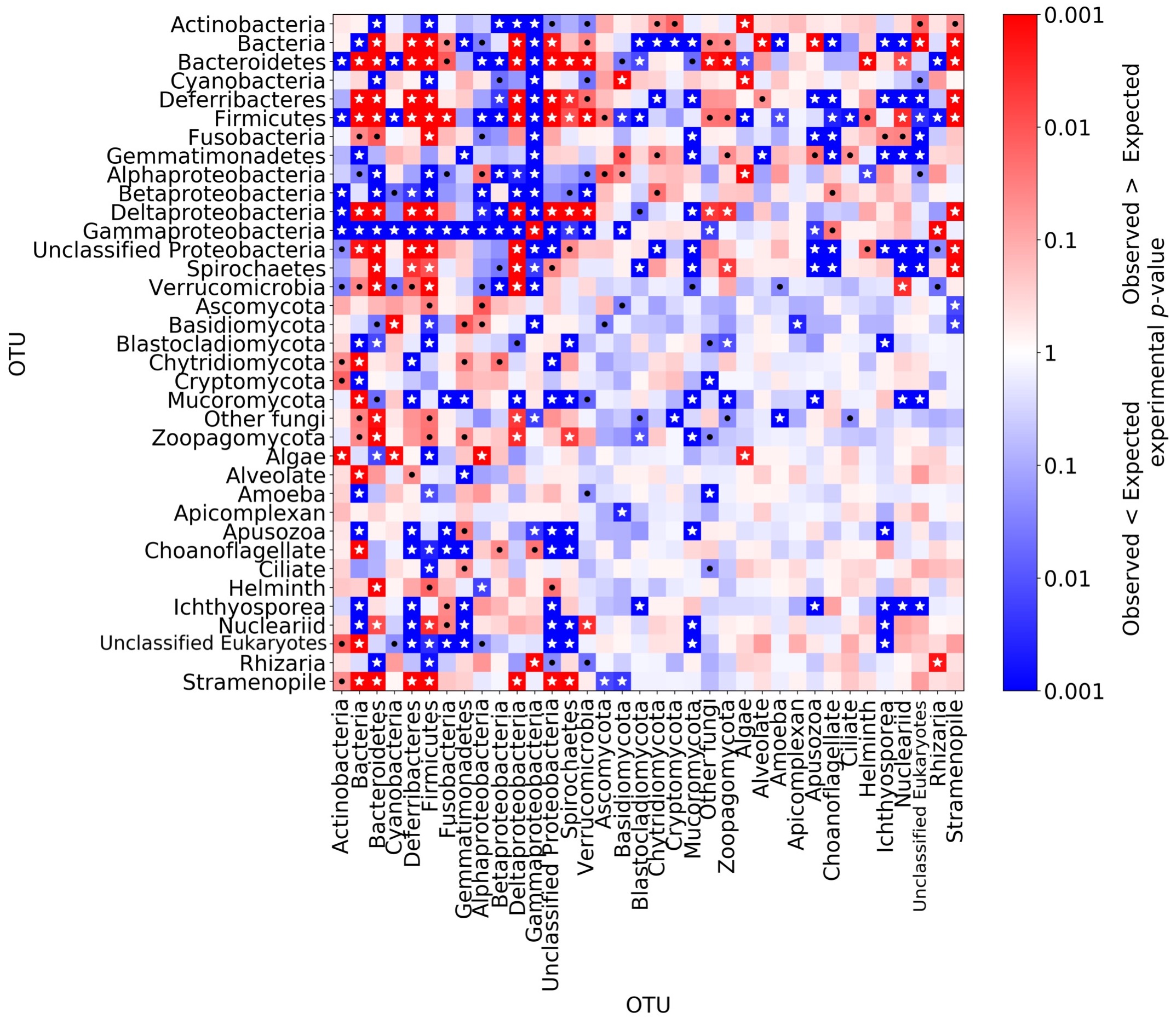

Supplement: Supplementary file 2 — Fig. S2 [file ECE3-11-9293-s005.jpg]

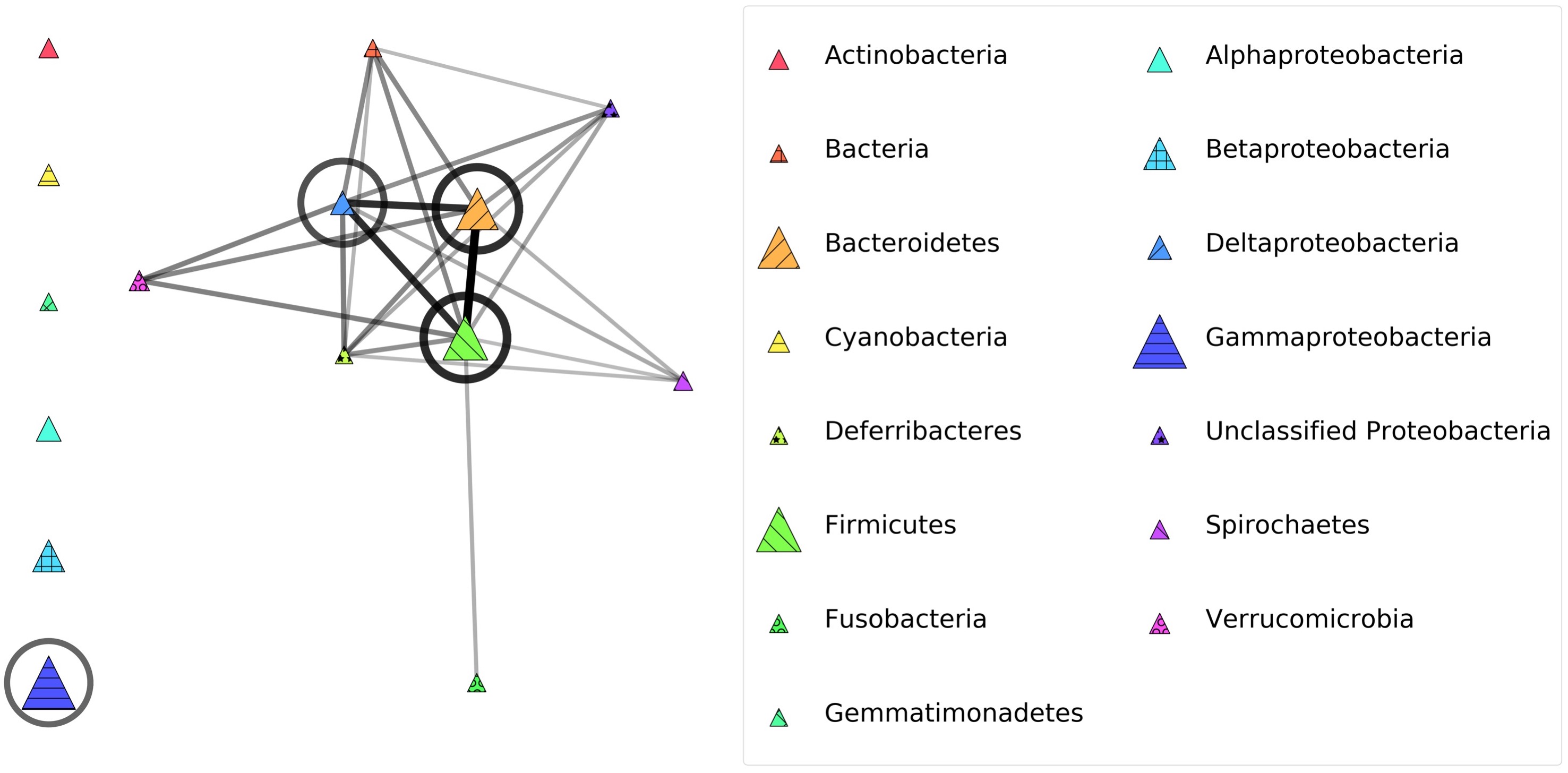

Supplement: Supplementary file 3 — Fig. S3 [file ECE3-11-9293-s001.jpg]

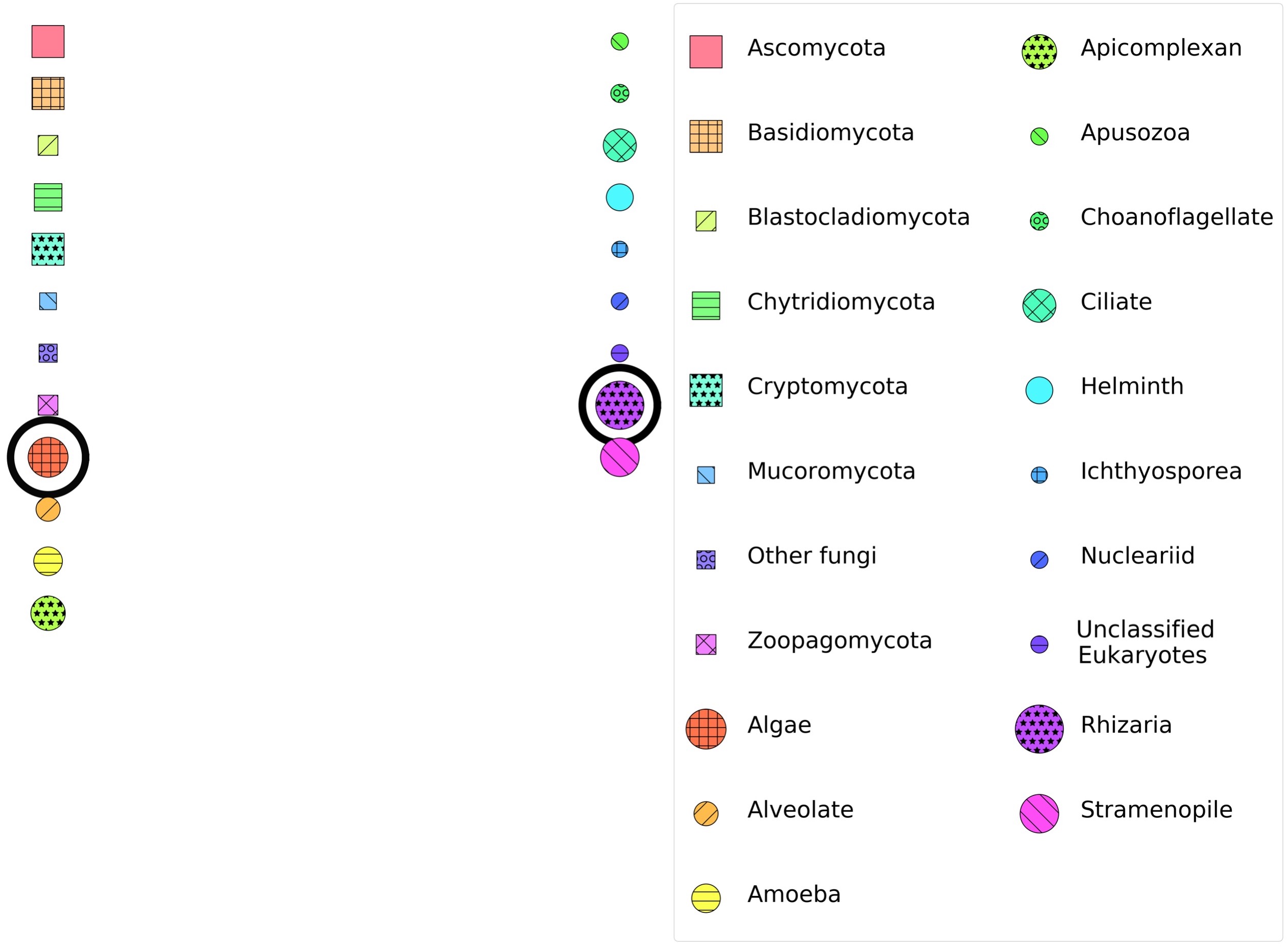

Supplement: Supplementary file 4 — Fig. S4 [file ECE3-11-9293-s004.jpg]

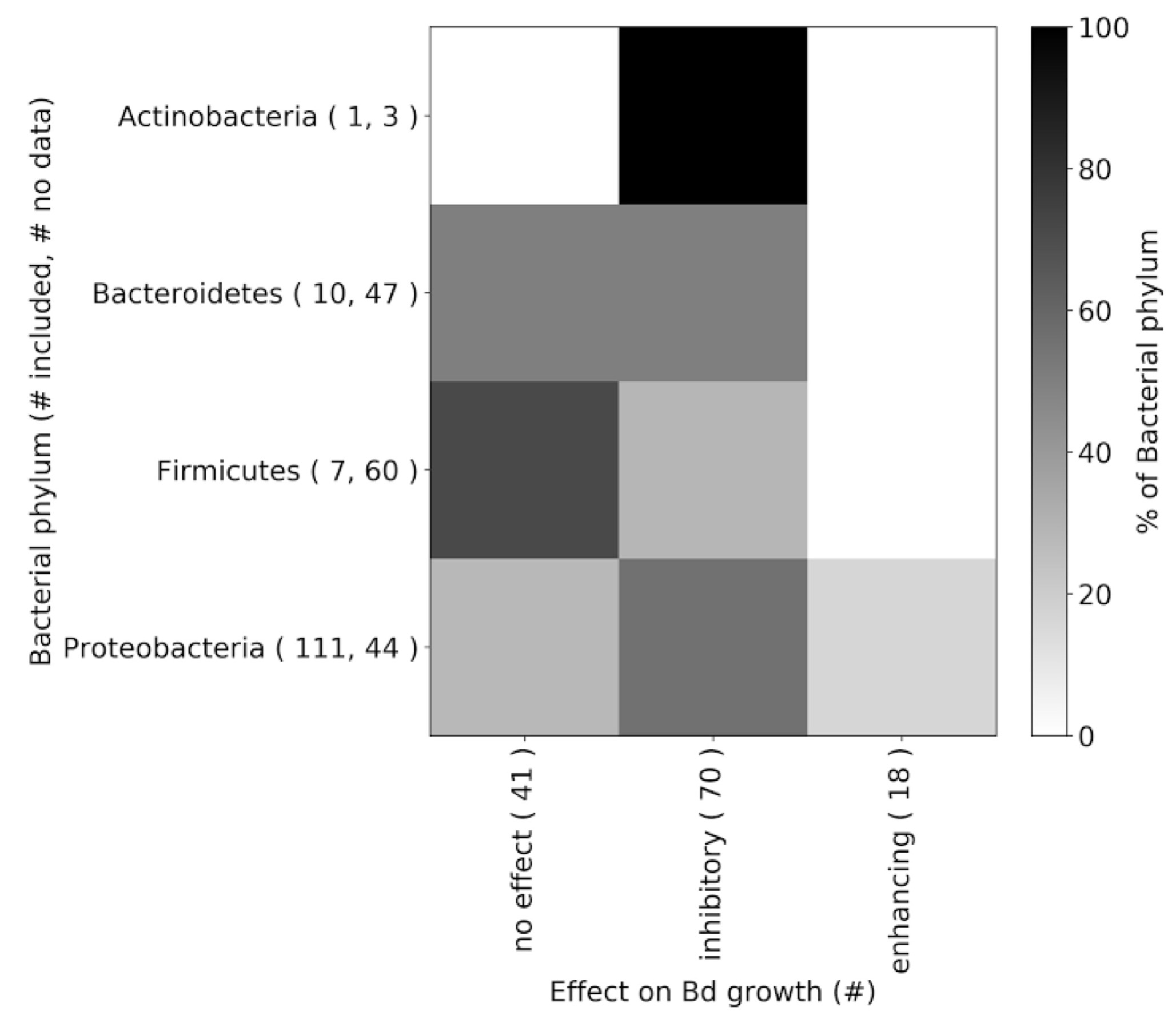

Supplement: Supplementary file 5 — Fig. S5 [file ECE3-11-9293-s003.jpg]
