# Supplementary material for: Geography, Host Genetics, and Cross‐Domain Microbial Networks Structure the Skin Microbiota of Fragmented Brazilian Atlantic Forest Frog Populations
Source: Ecol Evol. 2021 Jun 18;11(14):9293–307. doi: 10.1002/ece3.7594 (PMC8293785; doi:10.1002/ece3.7594)
Supplement: Supplementary file 6 — Supplementary materials [file ECE3-11-9293-s002.docx]

**SUPPLEMENTAL FIGURE LEGENDS**

**Figure S1.** Plots of observed OTUs retained across sequencing depth for 16S and 18S sequences across individuals (top panels) and number of samples retained across site types (bottom panels). Rarefaction thresholds (represented by dashed vertical red lines) of 2000 sequences for 16S and 1000 sequences for 18S were selected to strike a balance between a representative sample of OTUs and sufficient sample sizes across sites for statistical analyses.

**Fig. S2.** Heat map depicting co-occurrence between bacteria and microeukaryotes. The more saturated the red, the stronger the positive association between two taxa, and the more saturated the blue, the stronger the negative association. Black dots represent a significant deviation from random co-occurrence with p < 0.05, and white stars represent p < 0.01.

**Figure S3.** Microbiome network showing associations between bacterial OTUs. The size of the symbol indicates the relative abundance of each taxon by number of OTUs. Stronger associations are indicated by thicker/darker network branches between symbols (associations of OTUs among taxa) or circles around symbols (associations of OTUs within a taxon). Links were included between taxa which co-occurred significantly (a = 0.05) more often than in the null model. A circle around a taxon represents a self-edge. The edge weight was scaled according to the Z-score of the co-occurrence (observed - expected) / standard deviation, to the power of 0.5 to make the variation in score more visually clear.

**Figure S4.** Microbiome network showing associations between microeukaryotic OTUs. Fungi are represented by squares, and protists (non-fungal microeukaryotes) are represented by circles. The size of the symbol indicates the relative abundance of each taxon by number of OTUs. Stronger associations are indicated by thicker/darker network branches between symbols (associations of OTUs among taxa) or circles around symbols (associations of OTUs within a taxon). Links were included between taxa which co-occurred significantly (a = 0.05) more often than in the null model. A circle around a taxon represents a self-edge. The edge weight was scaled according to the Z-score of the co-occurrence (observed - expected) / standard deviation, to the power of 0.5 to make the variation in score more visually clear.

**Figure S5.** OTUs in bacterial phyla from *T. taophora* skin swabs that matched representative sequences from Woodhams et al. (2015). In parentheses next to the bacterial phyla on the y-axis are the number of OTUs that matched the database followed by the number of OTUs that did not have a match. In parentheses on the x-axis are the numbers of OTUs in total found in each group.
